# The status of scholarly efforts of librarians on health literacy: a bibliometric analysis

**DOI:** 10.5195/jmla.2022.1253

**Published:** 2022-04-01

**Authors:** Alexandria Quesenberry Wilson, Courtney Wombles, R. Eric Heidel, Kelsey Leonard Grabeel

**Affiliations:** 1 aqwilson@utmck.edu, Assistant Professor, Research and Learning Services Librarian, Preston Medical Library/Health Information Center, University of Tennessee Graduate School of Medicine, University of Tennessee Medical Center, Knoxville, TN; 2 courtney.wombles@lmunet.edu, Medical Librarian, Reed Health Sciences Library, Lincoln Memorial University, Knoxville, TN; 3 rheidel@utmck.edu, Associate Professor of Biostatistics, Department of Surgery, University of Tennessee Graduate School of Medicine, Knoxville, TN; 4 kgrabeel@utmck.edu, Associate Professor, Assistant Director, Preston Medical Library, Health Information Center, University of Tennessee Graduate School of Medicine, University of Tennessee Medical Center, Knoxville, TN

**Keywords:** health literacy, librarians, bibliometrics

## Abstract

**Objective:**

In order to determine the status of scholarly efforts on health literacy by librarians, researchers examined the characteristics of health literacy publications authored by librarians from 2000 to 2020.

**Methods:**

Bibliometric analysis was used to assess the indicators of productivity, affiliation, collaboration, and citation metrics of librarians in health literacy–related research. Data were collected using the Scopus database; articles were screened for inclusion before importation into Microsoft Excel for analysis. SPSS software was used to run basic descriptive statistics.

**Results:**

Of 797 search results, 460 references met the inclusion criteria of librarian authorship. There was a significant linear trend upward in publications since 2001 with an average increase of 1.52 papers per year. The number of publications per year peaked in 2019 (n=59). *Journal of Consumer Health on the Internet* was the most prolific journal. The majority of references were authored by at least two authors and by multidisciplinary teams. Nineteen percent (n=107) of the librarian authors were responsible for more than one publication, and 84.1% of publications were cited at least once.

**Conclusions:**

In the last two decades, librarian involvement in health literacy publications has exponentially increased, most markedly in the years following 2014. The productivity, multidisciplinary collaboration efforts, and consistent growth in literature indicate that librarians are engaged in health literacy scholarship. Further research is needed to explore the work of librarians whose impacts on health literacy may not be reflected within well-indexed, peer-reviewed publications.

## INTRODUCTION

Since it was first introduced, health literacy has evolved from focusing on an individual's ability to comprehend patient education into a broader understanding of the role of the health care system [1]. As a result of this evolution, Healthy People 2030 updated the previously accepted health literacy definition to include personal health literacy as well as organizational health literacy [2]. Over the years, health literacy research within many different disciplines has grown and evolved, including the discipline of library and information science [3]. Librarians are well poised to address health literacy due to their roles as researchers, teachers, and information professionals, yet there has been inadequate study on the intersection between health literacy and librarianship. The current state of health literacy knowledge and practice is well captured by Oelschlegel et al. and Barr-Walker, who note that librarians' experiences providing information to clinicians to guide clinical decision-making, as well providing access to consumer health information to patients, position them to contribute to the improvement of health literacy [4, 5].

One way to examine the intersection between health literacy and librarian scholarship is bibliometrics. Bibliometric analysis of quantitative publication metrics can provide a framework to examine various publication characteristics [6]. Few librarians have published on health literacy using a bibliometric methodology, but those who have define it as a new and increasing area of research [3]. Bankson presents the first and only comprehensive investigation of health literacy information, using bibliometric analysis to demonstrate a growing interest in health literacy as a research topic [3]. Nine databases were used in this research to analyze 643 articles published from 1997 to 2007. Bankson examined three indicators: growth of literature, core journals, and results by database. The bibliometric analysis with the selected articles revealed a clear upward trend in the number of health literacy articles published each year. The dramatic increase in articles from 1997 to 2007 showed a growing recognition of health literacy as a research topic, as well as a better understanding of the subject [3]. Another bibliometric analysis related to health literacy includes research by Kondilis et al., who presented a bibliometric study limited to PubMed and health literacy scholarship in the European Union [7]. The researchers' bibliometric analysis covered the period of 1991 to 2005, and it examined data for both the United States and countries within the European Union. The analysis found inequalities in published health literacy research, with the twenty-five European countries producing less than one-third of publications when compared to the number of publications by authors affiliated with the United States [7].

To determine the scholarly impact librarians may have had on health literacy, we examined the status and characteristics of health literacy publications authored by librarians from 2000 to 2020. To paint a more complete picture, a combination of characteristics representing the three bibliometric indicator types (quantity, performance, and structural connections) was selected to determine the growth of librarian health literacy research over time and various outputs related to quantity, citation impact, and additional details on where librarians were publishing and whom they were publishing with [8, 9]. Specifically, this study examines the following publication characteristics of health literacy literature authored by librarians:

- Growth of literature over time
- Author productivity
- Author institutional affiliation
- Publication country
- Journal productivity by type
- Citation impact
- Document access

## METHODS

Data for this study were collected using the Scopus (Elsevier) database, which provides a more multidisciplinary scope than the databases used in previous research. Library and information science–focused databases alone would not provide the important addition of health sciences journals, and health sciences and medical databases alone would not provide the library and information science scope needed for this study. Given that Scopus includes not only library and information science journals and medical journals, but journals outside of these fields as well, this database was selected to provide the widest net in terms of collecting data that would allow researcher analysis without machine learning. A retrospective search was performed in the database with dates limited from 2000 to 2020 and publication types limited to article, review, or conference paper. A focused search strategy was created using terms related to health literacy and libraries/librarianship. The health literacy terms *health literacy* and *health information literacy* were searched within quotation marks and using the title, abstract, and keyword field code (TITLE-ABS-KEY (“health literacy” OR “health information literacy”). For libraries/librarianship, terms related to libraries, librarians, and informationists were searched using the title, abstract, and keyword field code. Truncation was used with “librar-" and with informationist to capture plurals and librarians/libraries (TITLE-ABS-KEY (librar* OR informationist*). This was combined with the Boolean operator “OR” to an author affiliation search to identify authors working in libraries, information centers, or information science, with truncation used for librar- and information to capture those articles with a librarian author but not necessarily about research taking place in libraries (AFFIL (librar* OR information*). The search was run and completed on June 1, 2021.

The results were exported to Rayyan, a web application designed for systematic reviews, to screen for inclusion [10]. Screening was completed blindly by two of the authors, and any disputes were settled by a third researcher. The inclusion criteria were two-fold: at least one author must be a librarian and/or possess a library/information science degree, and the concept of health literacy must be included in the title, abstract, or keywords of the document record. For simplicity, those authors working in libraries or information centers and/or possessing a library/information science degree will be referred to as “librarians” in this paper. Additionally, author collaborations that include at least one librarian and at least one person outside the librarianship field will be referred to as “multidisciplinary.”

The bibliographic data for the included articles were exported to a Microsoft Excel spreadsheet. These data included article titles, author names, affiliations, year published, journal names, journal subject, citation counts, funding sources, and access level [11]. Due to database indexing inconsistencies, author name and affiliation data were cleaned manually.

Descriptive and frequency statistics (means and standard deviations for normally distributed variables; medians and interquartile ranges for non-normal data) were performed to describe the sample of published papers. Normality was checked using skewness and kurtosis statistics. Frequency and percentage statistics were used for categorical/binary variables. A simple linear regression model was employed to test for any trend in the number of published documents across time. A 95% confidence interval for the regression parameter associated with number of documents per year was calculated and plotted. The unstandardized beta coefficient, its respective standard error, and the 95% confidence interval were all reported and plotted visually. SPSS version 26 (Armonk, NY: IBM Corp.) was used to conduct all the statistical analyses, and statistical significance was assumed at an alpha value of 0.05 [12].

## RESULTS

Over n=16,000 documents indexed in the Scopus database were about health literacy from 2000 to 2020. The number of documents decreased to n=849 after adding in the librarianship search concepts, and this number was further reduced to n=797 with the addition of the article, review, and conference paper limiters. After screening, n=460 documents met the inclusion criteria and were included in this analysis.

### Growth over time

**Figure 1:**
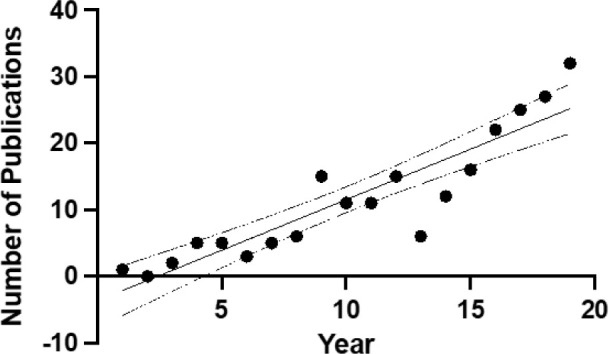
Linear regression of documents by year

Publications per year were varied but most markedly increased in the years following 2014 (Figure 1). There was a significant linear trend upward in documents since 2000, with documents increasing each year at 1.52 on average (*p*<0.001, B=1.52, SE=0.17, 95% CI 1.16–1.875). Documents per year peaked in 2019 with 59 documents. The largest increase occurred from 2014 (20 documents) to 2015 (34 documents).

### Authors

The 460 included documents were authored by 561 librarians. Solo librarian authors published 26.7% (n=123) of the documents. In terms of collaboration and coauthorship, of the 337 documents written by two or more authors, 36.5% (n=123) of documents were by all librarian author teams, which means the majority at 63.5% (n=214) were produced by a multidisciplinary team. The highest number of coauthors was 28 (three of these authors were librarians), and the average number of all authors per publication distributed across the documents was 3.35 (SD=2.82, median=3.00, IQR=1.00–4.00). Table 1 shows the most productive authors with ≥4 documents and their affiliations at the time of most recent publication. Nineteen percent (n=107) of the librarian authors had published more than one document.

**Table 1:** Most productive authors with ≥4 documents

Author	No. included documents	Affiliation
Hirvonen N	12	University of Oulu
Huotari ML	11	University of Oulu
Monkman H	10	University of Victoria
Xie B	10	University of Texas at Austin
Enwald H	8	University of Oulu
Grabeel KL	8	University of Tennessee Graduate School of Medicine
Jansen CJM	8	University of Groningen
Kushniruk A	8	University of Victoria
Keselman A	7	US National Library of Medicine
Oelschlegel S	7	University of Tennessee Graduate School of Medicine
St. Jean B	7	University of South Florida
Kim SU	6	University of Kentucky
Niemelä R	6	University of Oulu
Rubenstein EL	6	University of Oklahoma
Greene Taylor N	5	University of South Florida
Harnett S	5	University of Florida–Jacksonville
Kodama C	5	University of Maryland
Subramaniam M	5	University of Maryland
Chang YK	4	Higher Colleges of Technology
He Z	4	Florida State University
Hoeks J	4	University of Groningen
Koops van 't Jagt R	4	University of Groningen
Shipman JP	4	University of Utah
Smith CA	4	University of Wisconsin–Madison

### Affiliation

**Figure 2:**
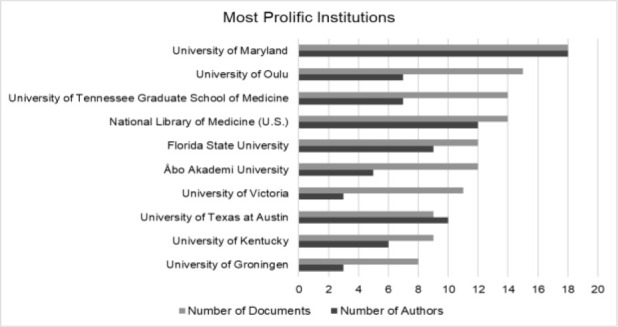
Number of documents and number of librarian authors by institutional affiliation

Librarian author affiliations were examined by their primary affiliation at the time of publication. The University of Maryland was the most prolific institution with n=18 articles and had the highest number of affiliated authors at n=18. With the exception of the US National Library of Medicine, the top institutions were all academic universities. Figure 2 shows the most prolific institutions with ≥8 documents and the number of affiliated librarian authors. Geographically, nearly half of the authors (48%, n=272) were affiliated with the United States, but overall the authors represented the following 48 countries in descending order based on productivity: United States, Canada, United Kingdom, Iran, Finland, Australia, Netherlands, China, Nigeria, South Korea, South Africa, Italy, Taiwan, Germany, India, Japan, Spain, Denmark, Pakistan, Singapore, Egypt, Hong Kong, Israel, Kenya, Kuwait, Malaysia, Namibia, Norway, Sweden, Zimbabwe, Belgium, Brazil, Congo, Croatia, Cuba, Ethiopia, Ghana, Greece, Iceland, New Zealand, Russian Federation, Slovenia, Sri Lanka, Switzerland, Tanzania, Thailand, Uganda, and the United Arab Emirates.

### Journals

The n=460 documents were distributed across 154 journals for an average of 2.99 documents per journal (SD=6.10, median=1.00, IQR=1.00–2.00). Sixty-one percent (n=94) of journals only had one included document. Journals covering library science, information science, or informatics based on their Scopus indexing are referred to as library journals in this paper. Overall, there were n=73 library journals included, and 77.8% (n=358) of the total documents were published within those journals. Table 2 shows the most prolific library journals, which are those with ≥5 documents.

**Table 2:** Most prolific library journals

Journal title	No. included documents (%)
*Journal of Consumer Health on the Internet*	55 (12)
*Health Information and Libraries Journal*	31 (6.7)
*Journal of Hospital Librarianship*	26 (5.6)
*Journal of the Medical Library Association*	26 (5.6)
*Journal of Medical Internet Research*	20 (4.3)
*Studies in Health Technology and Informatics*	15 (3.3)
*Proceedings of the Association for Information Science and Technology*	13 (2.8)
*Communications in Computer and Information Science*	11 (2.4)
*Reference Services Review*	10 (2.2)
*Journal of Health Communication*	9 (2)
*Medical Reference Services Quarterly*	9 (2)
*Information Research*	6 (1.3)
*Library Philosophy and Practice*	6 (1.3)
*Evidence Based Library and Information Practice*	5 (1.1)
*Library Trends*	5 (1.1)
*Libri*	5 (1.1)

Table 3 shows the most prolific nonlibrary journals out of the 81 included, which are those with ≥2 documents. Primary subject areas of these journals included medicine (n=55), computer science (n=8), nursing (n=7), social sciences (n=6), pharmacology (n=2), anthropology (n=1), engineering (n=1), and environmental science (n=1).

**Table 3:** Most prolific nonlibrary journals

Journal title	No. included documents
*Patient Education and Counseling*	4
*BMJ Open*	3
*Health Communication*	3
*International Journal of Environmental Research and Public Health*	3
*Academic Emergency Medicine*	2
*Association for Computing Machinery International Conference Proceeding Series*	2
*American Journal of Health Behavior*	2
*American Journal of Preventive Medicine*	2
*Asian Pacific Journal of Cancer Prevention*	2
*Asia-Pacific Journal of Public Health*	2
*BMC Geriatrics*	2
*Health Education Journal*	2
*Journal of Education and Health Promotion*	2
*Journal of General Internal Medicine*	2
*Knowledge Management and E-Learning*	2
*Lecture Notes in Computer Science*	2

### Review articles

Out of the included documents, 48 (10.4%) were review articles. These included 10 scoping reviews, 6 literature reviews, and 32 systematic reviews. The majority of the review articles (68.7%, n=33) were written by multidisciplinary teams that included at least one librarian, which means 15 were authored by all librarian teams.

### Citation impact

As of July 21, 2021, 84.1% (n=387) were cited at least once with an average of 12 citations, but the standard deviation of 25.17 shows that the citation counts are extremely skewed and have high kurtosis (median=4.00, IQR=1.00–11.00) or have large amounts of variation within the dataset. Table 4 shows the documents most highly cited at ≥100 citations. Open access is designated by an asterisk (*) in the table, and the librarian author is bolded.

**Table 4:** Most highly cited documents

Title	Year of publication	Authors	Author affiliation(s)	Cited by
Promoting health literacy*	2005	**McCray AT**	US National Library of Medicine	226
Low health literacy and evaluation of online health information: A systematic review of the literature*	2015	Diviani N, Van Den Putte B, **Giani S**, Van Weert JCM	University of Amsterdam	182
Risk factors and screening instruments to predict adverse outcomes for undifferentiated older emergency department patients: a systematic review and meta-analysis*	2015	Carpenter CR, Shelton E, **Fowler S**, Suffoletto B, Platts-Mills TF, Rothman RE, Hogan TM	Washington University	155
Health information literacy and competencies of information age students: results from the interactive online Research Readiness Self-Assessment (RRSA)*	2006	Ivanitskaya L, O'Boyle I, **Casey AM**, Ivanitskaya L	Central Michigan University	151
Social media use among patients and caregivers: a scoping review*	2013	Hamm MP, Chisholm A, Shulhan J, Milne A, Scott SD, **Given LM**, Hartling L	Charles Sturt University Wagga	144
Internet usage by low-literacy adults seeking health information: an observational analysis*	2004	Birru MS, Monaco VM, **Charles L, Drew H**, Njie V, Bierria T, Detlefsen E, Steinman RA	University of Pittsburgh	141
A systematic review of factors influencing older adults' decision to accept or decline cancer treatment*	2015	Puts MTE, Tapscott B, Fitch M, Howell D, Monette J, Wan-Chow-Wah D, Krzyzanowska M, Leighl NB, **Springall E**, Alibhai SM	University of Toronto	125
Medication adherence in older adults with cognitive impairment: a systematic evidence-based review	2012	Campbell NL, Boustani MA, **Skopelja EN**, Gao S, Unverzagt FW, Murray MD	Indiana University School of Medicine	122
Exploring digital divides: an examination of eHealth technology use in health information seeking, communication and personal health information management in the USA	2011	**Lustria MLA, Smith SA, Hinnant CC**	Florida State University	115
Health literacy in the eHealth era: a systematic review of the literature	2017	**Kim H, Xie B**	The University of Texas at Austin	109
Effects of an eHealth literacy intervention for older adults*	2011	**Xie B**	The University of Texas at Austin	109
Health information seeking, receipt, and use in diabetes self-management*	2010	Longo DR, Schubert SL, **Wright BA**, Lemaster J, Williams CD, Clore JN	Virginia Commonwealth University	109

### Open access

Nine of the 12 most highly cited documents were published open access. In total, 36.3% (n*=*167) of included documents were published open access at varying levels.

## DISCUSSION

The bibliometric analysis described in this article complements previous bibliometric studies while addressing some of the gaps. While Bankson and Kondilis et al. examined publications on health literacy overall, this bibliometric analysis integrates the involvement and authorship of librarians in the research. The presented study examines health literacy publications authored by librarians from 2000 to 2020, which covers years not included in Bankson's analysis of 1997–2007 [3] and Kondilis et al.'s analysis of 1991–2005 [7]. Also, this bibliometric analysis covers international publications, which differs from Kondilis et al.'s scope of scholarship in the European Union. It also examines additional bibliometric indicators not included in Bankson's study [7, 3]. In terms of librarians and health literacy, Barr-Walker's review examined library programming and initiatives related to health literacy, while this study focuses on librarian health literacy scholarship.

This analysis suggests that librarians are increasingly authoring manuscripts on health literacy, with the highest increase in the years after 2014. As noted by Bankson, the growing recognition of the importance of health literacy in the first decade of the 2000s may have led to the increase in publications from 1997 to 2007 [3]. For our analysis, the increase in publications could also correlate with the continued growth of the recognition of health literacy, as indicated by the addition of health literacy in the United States' Healthy People 2010 [13] and “health literacy” being added as a Medical Subject Heading (MeSH) term in 2010 [5]. Additionally, the rise in publications throughout 2010 and onward could also be due to the Affordable Care Act (ACA) that went into effect that year, which defines and addresses health literacy in four sections [14, 15]. Libraries were specifically called upon by the federal government to assist the public with navigating ACA, which may have led to further interest in health literacy within the profession [16]. Bankson partially attributes the rise in publications to the Medical Library Association's (MLA) creation of the MLA Health Literacy Task Force in 2002, as well as a sponsored health literacy teleconference in 2003 [3]. Internationally, the World Health Organization published *Health Literacy: The Solid Facts* in 2013, which emphasized the importance of policies to strengthen health literacy globally [17].

Over the 20 years examined, 561 librarians published 460 articles on health literacy, further indicating that librarians are involved in health literacy research. A majority of the publications (63.5%) were authored by a multidisciplinary team, showing collaboration between librarians and colleagues from various backgrounds. Additionally, eight out of the twelve most highly cited articles included a librarian on the team, but not as first author. The large number of articles within this dataset with librarian coauthors suggests that disciplines that deal with health information find librarians to be useful collaborators. Of the librarians who have published on health literacy, only 19% have published more than four times, with the most prolific author having twelve publications. More research may be needed to examine why only a few librarians publish multiple times.

As shown originally by Kondilis et al., this study also found the United States to be the most prolific country in health literacy publications [7]. Of the most prolific institutions, two institutions were from Finland, further justifying findings that Finland is one of the leaders on health literacy publications in the European Union [7]. Furthermore, since our research focused primarily on librarians, this indicates librarians in Finland are publishing on health literacy. The most prolific institutions publishing were all academic universities, except for the US National Library of Medicine (NLM). Publishing productivity at academic universities may be due to faculty status requirements or the potential dedicated time for research. Throughout the time period of this study, it should be noted that the regional offices for the Network of the National Library of Medicine were based in the University of Pittsburgh; University of Maryland, Baltimore; University of Utah; University of Washington; University of Colorado; University of Iowa; University of North Texas Health Science Center; University of California, Los Angeles; University of Massachusetts Medical School; the University of Illinois at Chicago; Houston Academy of Medicine; New York Academy of Medicine; and New York University. Given NLM's emphasis on improving health literacy in Goal 2 of their Strategic Plan, this may have influenced the number of documents published by authors affiliated with these institutions [18, 19].

The results show a higher number of nonlibrary journals than library journals, which again shows librarian roles on multidisciplinary teams. Bankson identified ten “core journals” of health literacy based on productivity [3]. Five of these journals were represented in this study, including *Journal of Health Communication* (Table 2) and *Patient Education and Counseling* (Table 3). This shows that librarians are capable of being valued research team members whose scholarly contributions are rigorous enough to be published within Bankson's core journals of health literacy. Of the nonlibrary journals, the most frequent Scopus-defined subject category was “medicine.” This provides additional insight into where librarians are publishing when their manuscripts are published outside the field of library and information science. Moreover, eight of the library journals were medical and/or health related. Although more documents were published in nonlibrary journals, the library journals had a higher number of documents per journal. This shows that there are ample opportunities for librarians to publish on health literacy within the field of library and information science. The decision of where to publish and why is complex, so additional research may be necessary to examine how librarians and their nonlibrarian collaborators make the decision to either publish within library journals or in nonlibrary journals.

One way to measure publication impact is through citation count. The most cited article found in this research was written by one author, a librarian. Nine out of the twelve articles most cited are open access, which could contribute to the high citation count as they are freely available and possibly more easily cited since they are more accessible [20]. Only four of the twelve articles have librarian-only authors. As seen in Table 4, five of the most highly cited documents are systematic reviews and one is a scoping review. This finding aligns with previous research, which finds that knowledge synthesis articles are more likely to be cited than other article types; it also demonstrates the central role that librarians can play on review teams [21, 22]. Reviews and other knowledge syntheses require great amounts of effort, but that effort isn't always acknowledged with authorship for librarians. There are likely many health literacy review articles with significant librarian involvement that were not counted in these results because the librarian was not listed as an author but rather mentioned in the acknowledgments or not mentioned at all. It is important to note that publication impact is complicated and varied. While authors cite papers for many reasons, citation counts don't necessarily align with quality [6]. Our results showed large variation within citation counts, which is not unusual. Citation counts, impact factor, and other similar arithmetic-based indicators have limitations but can be examined with other indicators for a fuller picture of a scholarly work.

There are several limitations to this study. Despite applying some systematic methodology during the screening process, this is not a systematic or comprehensive study of librarianship and health literacy. We searched one multidisciplinary database with a scope encompassing both the health literacy and library science fields to allow for richer details to be analyzed, but this excluded librarian contributions not indexed within Scopus. Selection bias might also be present due to the search strategy. Health literacy is complex, and the search used the term “health literacy,” which may not have been explicitly included in some articles as there are possible synonyms. Additionally, we did not review which specific subtopic or theme of health literacy the librarian was publishing on, but instead only examined if a librarian was an author and the journal subject. This limited the analysis on which field of librarianship was most prevalent in publishing on health literacy. As Carpenter et al. noted, “While publication metrics can provide compelling narratives, no single metric is sufficient for measuring performance, quality, or impact by an author” [9]. With this in mind, we selected indicators related to quantity, performance, and structural connections for a more complete narrative, but we acknowledge that author impact and research evaluation is not only demonstrated through bibliometric analysis. Furthermore, this analysis only reviewed the scholarly efforts by librarians that were published. There are likely many health literacy–related efforts that have been made by librarians that are not published, which would require further research.

## Data Availability

Data associated with this article are available in the Open Science Framework at https://doi.org/10.17605/OSF.IO/DMU2R.
